# Career interest and perceptions of nephrology: A repeated cross-sectional survey of internal medicine residents

**DOI:** 10.1371/journal.pone.0172167

**Published:** 2017-02-16

**Authors:** Michael N. Daniels, Sharon Maynard, Ivan Porter, Hope Kincaid, Deepika Jain, Nabeel Aslam

**Affiliations:** 1 Department of Medicine, Lehigh Valley Health Network, Allentown, Pennsylvania, United States of America; 2 Department of Medicine, Mayo Clinic College of Medicine, Mayo Clinic Florida, Jacksonville, Florida, United States of America; 3 Network Office of Research & Innovation, Lehigh Valley Health Network, Allentown, Pennsylvania, United States of America; Universidade Estadual Paulista Julio de Mesquita Filho, BRAZIL

## Abstract

**Background:**

Interest in nephrology careers among internal medicine residents in the United States is declining. Our objective was to assess the impact of the presence of a nephrology fellowship training program on perceptions and career interest in nephrology among internal medicine residents. A secondary objective was to identify commonly endorsed negative perceptions of nephrology among internal medicine residents.

**Methods:**

This was a repeated cross-sectional survey of internal medicine residents before (Group 1) and 3 years after (Group 2) the establishment of nephrology fellowship programs at two institutions. The primary outcome was the percentage of residents indicating nephrology as a career interest in Group 1 vs. Group 2. Secondary outcomes included the frequency that residents agreed with negative statements about nephrology.

**Results:**

131 (80.9%) of 162 residents completed the survey. 19 (14.8%) residents indicated interest in a nephrology career, with 8 (6.3%) indicating nephrology as their first choice. There was no difference in career interest in nephrology between residents who were exposed to nephrology fellows during residency training (Group 2) and residents who were not (Group 1). The most commonly endorsed negative perceptions of nephrology were: nephrology fellows have long hours/burdensome call (36 [28.1%] of residents agreed or strongly agreed), practicing nephrologists must take frequent/difficult call (35 [27.6%] agreed or strongly agreed), and nephrology has few opportunities for procedures (35 [27.3%] agreed or strongly agreed). More residents in Group 2 agreed that nephrology is poorly paid (8.9% in Group 1 vs. 20.8% in Group 2, P = 0.04), whereas more residents in Group 1 agreed that nephrologists must take frequent/difficult call (40.0% in Group 1 vs. 18.1% in Group 2, P = 0.02).

**Conclusions:**

The initiation of a nephrology fellowship program was not associated with an increase in internal medicine residents’ interest in nephrology careers. Residents endorsed several negative perceptions of nephrology, which may affect career choice.

## Introduction

Applications to U.S. nephrology subspecialty training programs has been declining for several years. In the 2009 National Residency Matching Program (NRMP) Match, there were 578 nephrology applicants, and 94.8% of posted positions were filled [1]. In the 2016 NRMP Match, there were only 298 applicants for 466 nephrology fellowship positions, and only 59.2% of posted positions were filled [2]. Efforts have focused on identifying factors discouraging residents from choosing nephrology, in order to reverse this trend and increase interest in nephrology careers [3–5]. Few studies have examined attitudes of internal medicine residents toward nephrology careers.

Studies in other fields have suggested that the presence of subspecialty trainees at an institution increases interest in that field among more junior trainees [6–8]. There is no data on the influence of the presence of nephrology training programs on residents’ career choices. The majority of nephrology fellows are satisfied with their decision [9]. The 2015 Nephrology Fellows Survey reported that 71.8% of fellows would recommend nephrology to medical students and residents [10]. We hypothesized that the presence of a nephrology fellowship program would increase IM residents’ interest in nephrology. Our objective was to assess the impact of the presence of nephrology fellows and nephrology training programs on internal medicine residents’ interest in nephrology, by surveying residents at two institutions before and after the initiation of nephrology fellowship training programs.

## Methods

This was a repeated cross-sectional survey of internal medicine residents at two academic medical centers which initiated nephrology fellowship training programs in 2012. Group 1 was surveyed at both institutions before the initiation of the nephrology fellowship programs. Group 2 was surveyed at both institutions 3 years after the initiation of the nephrology fellowship programs. Residents were invited to participate prior to or following educational conference sessions. Surveys were anonymous and voluntary. The study was approved by the Institutional Review Boards (IRB) at the Lehigh Valley Health Network, and at the Mayo Clinic Florida. The IRB approved the use of implicit informed consent, instead of written or verbal informed consent, by the study participants. Participants implicitly indicated their consent to participate by their act of submitting a completed survey. Participants who declined to participate did so by either (1) submitting a blank survey, or (2) not submitting a survey. Participant consent was documented by the receipt of a completed survey. The use of implicit informed consent was justified because the act of obtaining individual written or verbal informed consent would have compromised the anonymity of the participants’ decision to participate.

The survey was adapted from a previously validated survey of Australian medical trainees [11], modified based on the aims of the study. The survey included questions on demographics, career interests, perceptions of nephrology, and experiences affecting career choice (S1 and S2 Figs). Residents were asked to rank their top three career choices. Residents were asked to indicate if they agreed with several negative perceptions of nephrology using a 5-point Likert Scale (S2 Fig). They were asked to report the impact of several experiences potentially affecting career choice using a 3-point scale.

The primary outcome was the percentage of residents indicating that they wished to pursue nephrology as a career. Secondary outcomes included the percentage of residents who agreed with the negative perception statements of nephrology.

Descriptive statistics were presented for the variables overall and separated by Group. The Chi-square test for independence was used to assess differences in proportions between the two groups. The Fisher’s Exact Test was used when the expected cell counts were <5. Two-tailed p-values < 0.05 were considered statistically significant. Study investigators performed double data entry and cross checked the data for inconsistencies using SPSS. Statistical analyses were performed using SPSS and SAS version 9.3 (SAS Institute, Cary, NC). Pairwise deletion was used for missing data.

The primary outcome, career interest in nephrology, was evaluated as a dichotomous variable, grouping respondents indicating nephrology as their first, second, or third career choice into one category and respondents who did not into a second category.

To evaluate resident perceptions of nephrology, we collapsed the answer categories. Responses of “Strongly Agree” and “Agree” were combined into one group, “Strongly Disagree” and “Disagree” were combined into a second group, and “Neutral” comprised a third group.

## Results

162 internal medicine residents were invited to participate, and 131 completed the survey, for an overall response rate of 80.9%. Two respondents declined participation. Three respondents were missing data for the primary outcome and no more than 5 respondents were missing data for any one of the secondary outcomes.

Table 1 shows demographics and specialty interests of surveyed residents. There were no significant differences between Group 1 (residents surveyed before the establishment of the nephrology fellowship program) and Group 2 (residents surveyed after the establishment of the fellowship program) with regard to training year, gender, or proportion of international medical graduates.

**Table 1:** Demographics and career interests of surveyed residents.

	Group 1^1^	Group 2^2^	Overall	P-value for comparison(Group 1 vs Group 2)
**Residency year**				
PGY-1^3^	39.3%	34.3%	36.4%	0.43
PGY-2	35.7%	30.1%	32.6%	
PGY-3	25.0%	35.6%	31.0%	
**Gender (% Male)**	52.7%	64.4%	59.4%	0.18
**International Medical Graduates**	16.4%	17.8%	17.2%	0.83
**First choice specialty**				
Cardiology	20.0%	21.9%	21.1%	0.28
Hospitalist Medicine	18.2%	8.2%	12.5%	
Hematology/Oncology	10.9%	11.0%	10.9%	
Gastroenterology	5.5%	12.3%	9.4%	
Critical Care/Pulmonology	3.6%	12.3%	8.6%	
Primary Care/Internal Medicine	7.3%	9.6%	8.6%	
Endocrinology	12.7%	4.1%	7.8%	
Nephrology	7.3%	5.5%	6.3%	
Rheumatology	5.5%	6.9%	6.3%	
Infectious Disease	1.8%	5.5%	3.9%	
Other	5.5%	1.4%	3.1%	
Palliative Medicine	1.8%	1.4%	1.6%	
**Time 1**^**st**^ **choice interest**				
Before medical school	14.6%	16.4%	15.6%	0.33
1^st^/2^nd^ Year Med School	5.5%	13.7%	10.2%	
3^rd^/4^th^ Year Med School	38.2%	39.7%	39.1%	
Residency	41.8%	30.1%	35.2%	
**Time of Specialty Decision**				
Before Med School	11.1%	6.9%	8.7%	0.48
During Med School	22.2%	19.2%	20.5%	
During Residency	55.6%	53.4%	54.3%	
Not Certain of 1^st^ Choice	11.1%	20.6%	16.5%	
**Completed Nephrology Rotation as a Resident**	64.3%	42.5%	51.9%	**0.01**
**Completed Nephrology Rotation as a Student**	41.1%	38.4%	39.5%	0.75
**Presence of Fellowship Program Affected Choice of Residency**	42.9%	34.3%	38.0%	0.32

^**1**^**Group 1:** Residents surveyed before the initiation of a nephrology fellowship program at their institution

^**2**^**Group 2:** Residents surveyed 3 years after the initiation of a nephrology fellowship at their institution.

^3^PGY = Post-graduate year

In Group 1 and Group 2 combined, the most popular specialty choices were cardiology (21.1%), hospital medicine (12.5%), hematology/oncology (10.9%), and gastroenterology (9.4%). Overall, 6.3% of residents indicated that nephrology was their first choice. There were no statistically significant differences in first choice specialties between Group 1 and Group 2 (P = 0.28). There was no statistically significant difference between two groups in the percentage of residents who indicated nephrology as their first, second, or third career choice (P = 0.93).

Overall, most residents (64.9%) reported they first became interested in their chosen specialty prior to or during medical school. However, of the respondents who were certain of their first choice, the majority (65.1%) made their decision during residency. There were no differences between Group 1 and Group 2 regarding timing of specialty interest or decision. Significantly more residents in Group 1 vs. Group 2 had completed a nephrology rotation as a resident (64.3% vs. 42.5%, P = 0.01).

**Fig 1:**
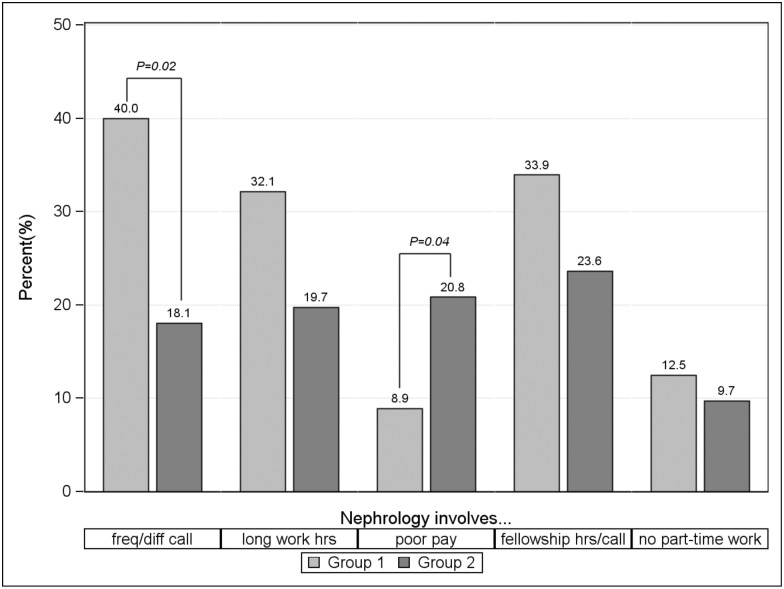
Negative perceptions of nephrology lifestyle factors among internal medicine residents. The percentage of residents who agreed or strongly agreed with negative lifestyle statements regarding nephrology are compared between Group 1 (residents who began residency prior to the establishment of a nephrology fellowship program) and Group 2 (residents who began residency after the establishment of a nephrology fellowship program).

To better understand residents’ perceptions of nephrology careers, we asked whether they agreed or disagreed with 12 negative statements about nephrology (Table 2). The most commonly endorsed negative perceptions of nephrology were lifestyle factors, including: “Nephrologists must take frequent/difficult call”, “Nephrologists have long work hours”, and “Nephrology fellowship requires long hours and burdensome night/weekend call.” Fig 1 compares residents’ perceptions of nephrology lifestyle factors in Group 1 vs. Group 2. Residents surveyed before the establishment of a nephrology fellowship (Group 1) were more likely to agree or strongly agree with the statement, “Nephrologists must take frequent/difficult call” compared to residents surveyed after the establishment of a nephrology fellowship program (Group 2) (40.0% vs. 18.1%, P = 0.02). However, residents in Group 2 were more likely to agree or strongly agree with the statement, “Nephrology is poorly paid” compared with residents in Group 1 (20.8% vs. 8.9%, P = 0.04). There were no statistically significant differences between Group 1 and Group 2 in the other negative perceptions of nephrology.

**Table 2:** Negative perceptions of nephrology among internal medicine residents.

Perception	Agree orstrongly agree
Nephrology fellowship requires long hours and burdensome night/weekend call	28.1%
Nephrologists must take frequent/difficult call	27.6%
Nephrology provides few opportunities for procedures	27.3%
Nephrology has long work hours	25.2%
Renal pathophysiology is too complex	23.8%
Medical school poorly prepared me to care for renal patients	23.0%
I have not done a rotation or managed many nephrology patients	19.1%
Nephrology is poorly paid	15.6%
Nephrology does not allow for part-time work	10.9%
I have not been exposed to any encouraging (positive) role models/mentors in nephrology	9.5%
The topic of nephrology is not interesting	6.3%

For each statement, residents were asked to choose among the following: strongly agree, agree, neutral, disagree, strongly disagree. Percentages are for all surveyed residents (Group 1 + Group 2).

Negative perceptions of nephrology were reported by a minority of residents who indicated nephrology as their specialty of choice. Of the 8 residents who indicated nephrology as their first choice specialty, 1 agreed with the statement that renal pathophysiology is too complex; 3 agreed that nephrologists have long work hours; 1 agreed that nephrology does not allow for part-time work; 2 agreed that nephrologists must take frequent/difficult call; and 1 agreed that nephrology fellowship requires long hours and burdensome night/weekend call.

Table 3 shows the reported impact of various experiences on residents’ career choice. Overall, residents stated that mentors/role models had the greatest impact on their career decision. Rotations during residency and medical school also impacted career decisions for many residents. Didactics during medical school had the least impact on career choice. A substantial number of residents (28.4%) reported that interactions with subspecialty fellows had a high impact on career choice. However, there was no significant difference between Group 1 and Group 2 with regard to the impact of this or any other experiences. Among the 8 residents who indicated nephrology as their first career choice, one did not answer questions regarding factors influencing their career choice, and 6 reported that rotations during medical school, residency, and mentors/role models had a high impact on their career decision. Fewer of these residents cited medical school didactics (3/7), personal/family experiences with disease (0/7), and interaction with subspecialty fellows (2/7) as having a high impact on their career choice.

**Table 3:** Experiences impacting career decisions among internal medicine residents. For each statement, residents were asked to indicate whether that experience had little, moderate, or high impact on their career decision. There were no significant differences between Group 1 and Group 2 (p>0.05 for all comparisons).

Experience	Residents reporting “high impact”		
	Group 1	Group 2	Overall
Mentors/Role models in specific career field	69.1%	73.6%	71.7%
Rotations during residency	56.4%	57.8%	57.1%
Rotations during 3^rd^/4^th^ year of medical school	47.3%	43.1%	44.9%
Interactions with subspecialty fellows	27.3%	29.2%	28.4%
Personal/family experience with disease illness	25.5%	16.7%	20.5%
Didactics during medical school	16.4%	8.3%	11.8%

## Discussion

In this study, we surveyed IM residents regarding their interest in nephrology careers and perceptions of nephrology before and after the establishment of nephrology fellowship programs at two institutions. We found that the presence of a nephrology fellowship program had no effect on interest in nephrology careers among IM residents; however it was associated with changes in some negative perceptions of nephrology. Long work hours, burdensome night/weekend call, and few opportunities for procedures were the most frequently cited negative perceptions of nephrology.

Studies in other fields have suggested that the presence of fellows or a fellowship program positively influences resident choice of subspecialty training. Lorin *et al* surveyed 178 IM residents from three academic medical centers about their interest in pulmonary and critical care medicine (PCCM) subspecialty training [6]. Several factors were associated with higher resident interest in PCCM training, including observing a high sense of satisfaction among PCCM fellows and receipt of encouragement from PCCM fellows about joining the field. Focus group data from Canadian residents suggests that lack of exposure to fellows may negatively impact a resident’s decision to apply to a specialty [7]. Among obstetrics and gynecology residents, the presence of a gynecologic oncology subspecialty training program at a primary teaching hospital was associated with a 4-fold higher resident career interest in that subspecialty [8].

There are several possible reasons we did not find a positive impact of fellowship on nephrology career interest. Our pre/post survey design did not allow us to control for temporal changes affecting our primary outcome. Our survey was administered in 2012 and again in 2015; over this period, there was a large decline in the number of U.S. nephrology fellowship applications via the NRMP [1]. Thus, a positive effect of fellowship programs may have been masked by the widespread decline in nephrology interest during the study period. Although fellows may impact a resident’s perceptions of nephrology in some positive ways, there was also suggestion of some negative impact on perceptions, particularly with regard to nephrologist salaries (see Fig 1).

Our findings provide insight into negative perceptions of nephrology among IM residents which may be contributing to the decline in nephrology career interest in the U.S. Most frequently cited negative perceptions relate to poor work/life balance and financial reimbursement, intellectual complexity, and lack of procedures. Over 25% of residents agreed that nephrologists and nephrology fellows have long work hours and frequent, burdensome night and weekend calls. Even among residents indicating nephrology as their first career choice, 3 of 8 (37.5%) agreed that nephrologists have long work hours. These perceptions probably reflect reality. Although nephrologists’ work hours and call may be similar to some more popular subspecialties, such as cardiology and hematology-oncology, nephrologists’ compensation ($277,499 according to the 2014 AGMA survey) is far less than these fields ($383,117 for non-invasive cardiology, $350,268 for hematology-oncology) [12, 13]. Compensation in nephrology is similar to that for hospital medicine ($241,250) or rheumatology ($240,250)–fields where work/life balance is more favorable than nephrology.

The emergence of hospital medicine as an appealing career path may be contributing to the decrease in interest in nephrology and other non-interventional hospital-based specialties. Hospital medicine was the second most popular career choice among our surveyed residents. Starting salaries for hospitalists rival those for nephrologists, with better work hours and no need for fellowship training. Whether the decline in nephrology trainees will eventually lead to a shortage of nephrologists and an increase in nephrologists’ compensation is unknown [10].

Exposure to a field as a trainee may increase interest in that field; for example, in the Lorin study, residents who had spent more time in the intensive care unit were more likely to be interested in pulmonary/critical care fellowship [6]. Rotations during residency were cited as one of the most important experiences impacting career decisions in our study (Table 3). Among surveyed residents who indicated nephrology as their first career choice, almost all (6/8) cited rotations during medical school and residency as having a high impact on their career decision. Thus, it is notable that only 51.9% of our surveyed residents reported completing a nephrology rotation as a resident, and only 39.5% completed a nephrology rotation as a medical student (Table 1). Working with IM residency programs to increase resident exposure to nephrology may increase interest in nephrology careers.

Time on specialty rotations can lead to mentoring relationships. Relationships with mentors and role models was the most important experience impacting career decisions among residents in our survey, including those choosing nephrology. Other studies have confirmed the importance of mentors in guiding career decisions [4, 14]. In our study, very few residents (9.5%) felt they lacked encouraging mentors in nephrology. Nephrology educators should continue to seek out mentoring roles and relationships with students and residents.

Some students and residents may be dissuaded from nephrology due to the complexity and intellectual challenge of the field. IM non-nephrology subspecialty fellows cited the complexity of renal pathophysiology and lack of procedures as reasons for not choosing nephrology [15]. In another study, 78% medical students felt that renal pathophysiology was too complex, irrelevant, or not interesting [3]. In our study, 23.8% of residents agreed that renal pathophysiology is “too complex.” A lack of mastery may feed the perception of complexity: 23% of our surveyed residents felt that medical school prepared them poorly to care for renal patients. Nephrology educators should focus on effective teaching strategies to improve student and resident confidence and sense of mastery [16, 17].

The perception that nephrology lacks procedures is not surprising because many nephrologists have ceded dialysis catheter placement and renal biopsy procedures to other specialties [18]. Whether diminishing opportunities for procedures are contributing to the decline in nephrology applications is unknown.

A strength of this study is the repeated cross-sectional study design, whereby two different groups of IM residents were surveyed at the same institutions, which helped control for institutional factors that may have affected responses. The response rate was high (80.9%), which increases confidence in survey results. Limitations included the inability to account for other factors that may have led to decreased interest in nephrology over time. It is unknown whether these findings are generalizable to non-U.S. internal medicine residents.

The field of nephrology is in crisis, and we need to identify and address the negative perceptions that deter residents from nephrology careers. This study demonstrates that negative perceptions of lifestyle factors in nephrology are widely held by IM residents. Efforts to improve interest in nephrology should focus on addressing these negative perceptions.

## Conclusions

We found no change in nephrology career interest among IM residents following the initiation of nephrology fellowship programs at two institutions. Residents’ most commonly endorsed negative perceptions were that nephrology has poor work/life balance, few opportunities for procedures, and that renal pathophysiology was too complex. Efforts to improve interest in nephrology should focus on addressing these negative perceptions and realities.

## Supporting information

S1 Fig Survey instrument, page 1. (DOC)

S2 Fig Survey instrument, page 2. (DOC)

S1 File Dataset. (XLSX)
