# Supplementary material for: Career interest and perceptions of nephrology: A repeated cross-sectional survey of internal medicine residents
Source: PLoS One. 2017 Feb 16;12(2):e0172167. doi: 10.1371/journal.pone.0172167 (PMC5313227; doi:10.1371/journal.pone.0172167)
Supplement: S1 Fig — (DOC) [file pone.0172167.s001.doc]

Resident Career Choice Survey

The purpose of this survey is to better understand what factors influence internal medicine resdients’ decision to pursue a particular subspecialty. The survey is voluntary. Both your decision to participate and your responses are anonymous: your answers cannot be linked back to your name or identity. The survey takes less than 10 minutes to complete. If you agree to particpate, please complete the survey, place it in the attached envelope, and return it to Mike Daniels or Deepika Jain. To opt-out, please place the blank survey in the provided envelope and return it to us.

***Please complete this survey once and only once.***

1. What is your current year of residency?

- PGY-1
- PGY-2
- PGY-3

1. Are you:

- Male
- Female

1. Are you an International Medical Graduate?

- Yes
- No

1. What specialty do you wish to pursue after residency? *Number up to three, in order of preference, with your first choice as # 1.*

___ Cardiology

___ Critical Care/Pulmonology

___ Endocrinology

___ Infectious Disease

___ Gastroenterology

___ Geriatrics

___ Hematology/Oncology

___ Hospitalist Medicine

___ Nephrology

___ Palliative Medicine

___ Primary Care/Internal Medicine

___ Rhuematology

___ Other (specify: _____________)

1. When did you first become interested in your #1 specialty choice?

- Before medical school
- During 1^st^/2^nd^ year medical school
- During 3^rd^/4^th^ year medical school
- During residency

1. If you are certain of your 1^st^ choice, when did you make this decision?

- Before medical school
- During medical school
- During residency
- Not certain of 1^st^ choice

1. Have you completed your residency nephrology rotation yet?
   - Yes
   - No
2. Did you complete a nephrology rotation as a medical student?

- Yes
- No

1. Was your choice of residency program affected by the presence of a specific fellowship program here?
   - Yes
   - No
