# Supplementary material for: Career interest and perceptions of nephrology: A repeated cross-sectional survey of internal medicine residents
Source: PLoS One. 2017 Feb 16;12(2):e0172167. doi: 10.1371/journal.pone.0172167 (PMC5313227; doi:10.1371/journal.pone.0172167)
Supplement: S2 Fig — (DOC) [file pone.0172167.s002.doc]

9. We are interested in your **perceptions** of nephrology and the **impact** they have on your career choice. To what extent do you agree with the following statements and what impact they have had on your career decision? Mark the most appropriate box for each statement.

| **Statements about Nephrology** | **Perceptions of Nephrology**  **CHOOSE ONE for each statement** | | | | | **Impact on Career Choice**  **CHOOSE ONE for ea stmt** | | |
| --- | --- | --- | --- | --- | --- | --- | --- | --- |
|  | **Strongly**  **Disagree** | **Disagree** | **Neutral** | **Agree** | **Strongly Agree** | **Little Impact** | **Moderate**  **Impact** | **High Impact** |
| **The topic of nephrology is not interesting.** |  |  |  |  |  |  |  |  |
| **Managing nephrology patients is unappealing because of the chronic nature of their disease** |  |  |  |  |  |  |  |  |
| **Renal pathophysiology is too complex.** |  |  |  |  |  |  |  |  |
| **Medical school poorly prepared me to care for renal patients** |  |  |  |  |  |  |  |  |
| **I have not been exposed to any encouraging (positive) role models/mentors in nephrology.** |  |  |  |  |  |  |  |  |
| **I have not done a rotation or managed many nephrology patients.** |  |  |  |  |  |  |  |  |
| **Nephrology is poorly paid** |  |  |  |  |  |  |  |  |
| **Nephrology provides few opportunities for procedures.** |  |  |  |  |  |  |  |  |
| **Nephrology has long work hours.** |  |  |  |  |  |  |  |  |
| **Nephrology does not allow for part-time work.** |  |  |  |  |  |  |  |  |
| **Nephrologists must take frequent/difficult call.** |  |  |  |  |  |  |  |  |
| **Nephrology fellowship requires long hours and burdensome night/weekend call.** |  |  |  |  |  |  |  |  |

10. We are interested in how the following **experiences** have affected your career decision. Mark the most appropriate box for each experience

| **Experiences Affecting Career Choice** | **Impact on Career Choice** | | |
| --- | --- | --- | --- |
|  | **Little Impact** | **Moderate Impact** | **High Impact** |
| **Didactics during medical school.** |  |  |  |
| **Rotations during 3^rd^/4^th^ year medical school.** |  |  |  |
| **Mentors/Role models in specific career field.** |  |  |  |
| **Rotations during residency.** |  |  |  |
| **Personal/family experience with disease illness.** |  |  |  |
| **Interactions with subspecialty fellows** |  |  |  |
